# Genetic Regulation of Mycotoxin Biosynthesis

**DOI:** 10.3390/jof9010021

**Published:** 2022-12-22

**Authors:** Wenjie Wang, Xinle Liang, Yudong Li, Pinmei Wang, Nancy P. Keller

**Affiliations:** 1 School of Food Science and Biotechnology, Zhejiang Gongshang University, Hangzhou 310018, China; 2 Institute of Food Biotechnology, Zhejiang Gongshang University, Hangzhou 310018, China; 3 Ocean College, Zhejiang University, Zhoushan 316021, China; 4 Department of Medical Microbiology and Immunology, University of Wisconsin-Madison, Madison, WI 53706, USA; 5 Department of Bacteriology, University of Wisconsin-Madison, Madison, WI 53706, USA

**Keywords:** mycotoxin, aflatoxin, patulin, citrinin, trichothecene, fumonisin, regulatory mechanism

## Abstract

Mycotoxin contamination in food poses health hazards to humans. Current methods of controlling mycotoxins still have limitations and more effective approaches are needed. During the past decades of years, variable environmental factors have been tested for their influence on mycotoxin production leading to elucidation of a complex regulatory network involved in mycotoxin biosynthesis. These regulators are putative targets for screening molecules that could inhibit mycotoxin synthesis. Here, we summarize the regulatory mechanisms of hierarchical regulators, including pathway-specific regulators, global regulators and epigenetic regulators, on the production of the most critical mycotoxins (aflatoxins, patulin, citrinin, trichothecenes and fumonisins). Future studies on regulation of mycotoxins will provide valuable knowledge for exploring novel methods to inhibit mycotoxin biosynthesis in a more efficient way.

## 1. Introduction

Mycotoxins are toxic secondary metabolites (SMs) widespread in filamentous fungi, particularly *Aspergillus*, *Penicillium*, *Monascus* and *Fusarium* genera, and represent a major threat to human and animal health (e.g., carcinogenicity, nephrotoxicity) [1,2,3,4]. Due to the toxicities, regulatory organizations have established maximum permissible levels for mycotoxins in food products in many countries. For example, the European Union (EU) has established a maximum content of 50 μg/kg of patulin (PAT) for apple-based juices, 25 μg/kg of PAT for solid food products, and 10 μg/kg of PAT for baby foods [5].

The control of mycotoxin contamination is based on two strategies: prevention of mycotoxin production and detoxification [6]. Chemical fungicides (e.g., tebuconazole, metconazole) and deploying disease-resistant plants are the main approaches for preventing pre-harvest plant infections by mycotoxin producing species [7]. Considering the safety issue of fungicide, biocontrol methods are proposed as alternatives by using living organisms against the growth of mycotoxin producing fungi [8]. Post-harvest contamination is largely prevented by controlled environments such as low humidity, hermetic packaging technology or artificial atmospheres [9,10]. Physical, chemical and biological techniques have been largely used to detoxify mycotoxins [11,12]. Absorbents are employed to physically remove mycotoxins, and chemical reaction exerts degradation effects toward mycotoxins [13]. Nevertheless, through efforts spanning several decades, mycotoxin decontamination methods still have many limitations. For example, current methods with fungicides have the problem of safety issue, short effective time, and fungicide resistance [7]. Detoxification methods cause nutrient loss, and are time-consuming and expensive, etc. [6]. As such, there is a great need for more effective approaches to manage mycotoxin contamination.

One of the new strategies is to discover specific mycotoxin-production inhibitors, which do not affect fungal growth but could control mycotoxin without incurring rapid spread of resistant fungal strains. For example, antimicrobial proteins and peptides (AMPs) with antifungal activity is a promising approach with low concentration which inhibits mycotoxin production by affecting its regulatory mechanism [14]. Therefore, a full understanding of regulatory mechanisms of mycotoxin biosynthesis could offer real opportunities to develop more effective management for mycotoxin contamination. In the past two decades, efforts have been made to characterize the biosynthesis of mycotoxins and their genetic regulation. This review presents our current knowledge of regulatory mechanisms of mycotoxin synthesis including environmental signals and genetic regulators.

## 2. Critical Mycotoxins

**Figure 1:**
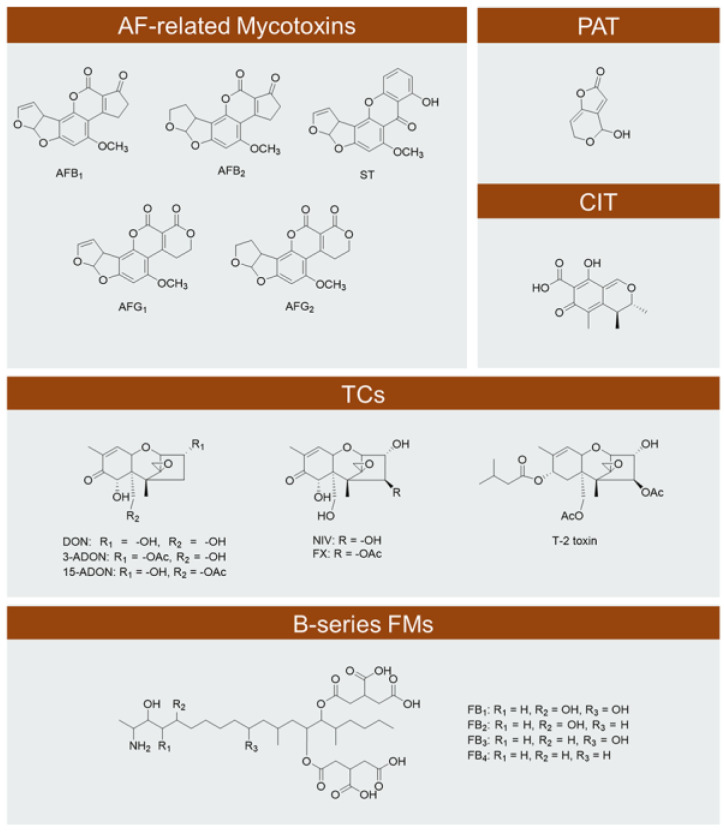
The most critical mycotoxins in food industry.

The most important mycotoxins include aflatoxins (AFs), AF-related sterigmatocystin (ST), PAT, citrinin (CIT), trichothecenes (TCs), and fumonisins (FMs) (Figure 1). The main producing species of these mycotoxins are listed in Table 1. Four major AFs (AFB_1_, AFB_2_, AFG_1_ and AFG_2_) and ST, which is the penultimate precursor of AFs [15], share the same polyketide pathway. PAT and CIT are also polyketide-derived mycotoxins. TCs are a large family of sesquiterpenoid secondary metabolites, and are defined by their heterocyclic structure including a 9,10-double bond and a 12,13-epoxide [7]. The *Fusarium* TCs of the greatest concern are deoxynivalenol (DON), acetylated DON (ADON), nivalenol (NIV), fusarenon X (FX) and T-2 toxin (Figure 1). FMs are polyketide-derived mycotoxins containing two tricarballylic acid side chains and one or more hydroxyl groups. B-series FMs are the most common among the four series (A, B, C and P), with fumonisin B_1_ (FB_1_) being the predominant and most toxic member, followed by fumonisin B_2_ (FB_2_), fumonisin B_3_ (FB_3_) and fumonisin B_4_ (FB_4_) (Figure 1) [16].

The biosynthetic pathways of these mycotoxins have been characterized well and the reader is referred to other reviews for details on the biosynthetic pathways [26,27,28,29]. In the following section, we summarize the regulators that control the biosynthesis of these mycotoxins.

## 3. Regulation Mechanism of Mycotoxin Biosynthesis

Regulation of mycotoxin biosynthesis is a complex process with various environmental factors forming a hierarchial regulatory network, including pathway-specific regulators, global regulators and epigenetic modification [30] as summarized in Table 2.

### 3.1. Pathway-Specific Regulator

The genes involved in the biosynthesis of mycotoxin are typically arranged in a biosynthetic gene cluster (BGC), containing not only synthases and/or synthetases genes but also many tailoring enzymatic encoding genes [73]. The cluster usually contains a pathway-specific regulator when the BGC contains more than five genes [74], and most of these transcription factors (TFs) function as positive regulators to induce expression of the remaining cluster-genes for the biosynthesis of final products. Indeed, all the mycotoxin BGCs discussed in this review contain the pathway-specific regulators for the activation of other genes in the BGC.

**Figure 2:**
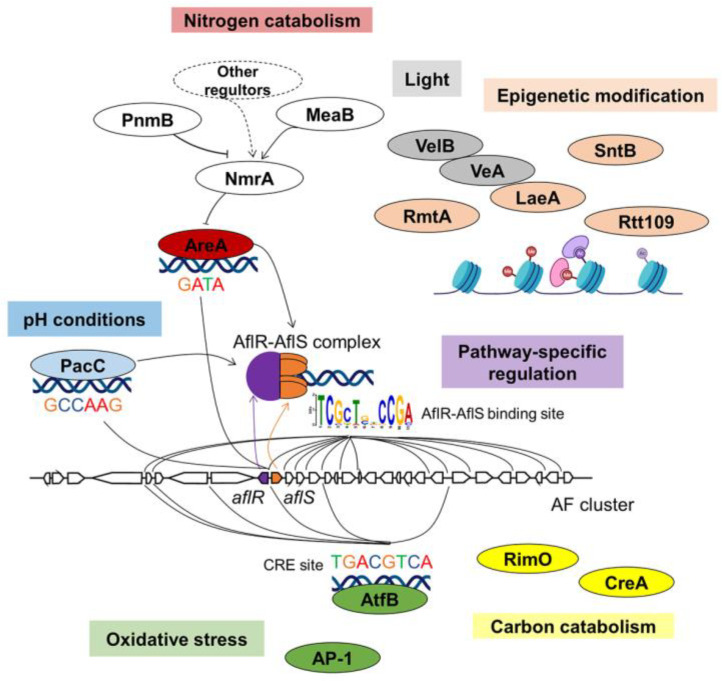
Regulatory mechanism of AF biosynthesis.

The AFs and ST are produced by the same biochemical pathway, and their gene cluster has been widely studied in *A. flavus*/*A. parasiticus* and *A. nidulans*. The AF/ST BGC includes ~30 genes and including two pathway-specific regulators AflR and AflS (previously named AflJ) (Figure 2) [31,32]. AflR is a Zn(II)_2_Cys_6_ type TF, which is only found in the fungal kingdom [75]. AflS is a TF without a conserved domain but forms a protein complex with AflR (1AflR + 4AflS), so AflS is often termed as a co-activator [32,76]. The expressions of both AflR and AflS need to meet the requirement of the proper ratio of AflS to AflR (~4:1) for the formation of a functional transcriptional activation complex (Figure 2). Then AflR/AflS complex binds to promoter regions of ST genes in *A. nidulans* by recognizing the palindromic pattern 5′-TCG(N_5_)CGA-3′ [77]. In *A. parasiticus*, in addition to 5′-TCG(N_5_)CGA-3′, the AflR/AflS complex is reported to also bind to 5′-TCGCAGCCCGG-3′ and a site with only 7-bp of the 5′-TCG(N_5_)CGA-3′ motif in the intergenic region of *aflR* and *aflS*, albeit with weak affinity [78]. The preferred binding sequence was found to be 5′-TCGSWNNSCGR-3′ (S = G or C, W = A or T, R = G or A, N = A or G or C or T). In *A. flavus*, the AflR binding site in the genome was identified by ChIP-Seq, which is an 18-bp palindromic sequence 5′-**CSSGGGWTCGA**WCCCSSG-3′ [79]. Positions 8–18 of this DNA motif are similar to the previously identified AflR/AflS complex binding sites, which suggest that they are motif A (underlined), while positions 1–11 constitute motif B (**bold**). AflR probably binds to either or both of motif A and motif B [79]. The abnormal expression of either *aflR* or *aflS* would reduce the concentration of a functional regulatory complex, then lower the ability to activate the expression of AF/ST biosynthetic genes and the production of AF/ST mycotoxins. Deletion of *aflR* abolishes AF/ST synthesis, and deletion of *aflS* results in a failure to convert intermediates to aflatoxin [80,81].

**Figure 3:**
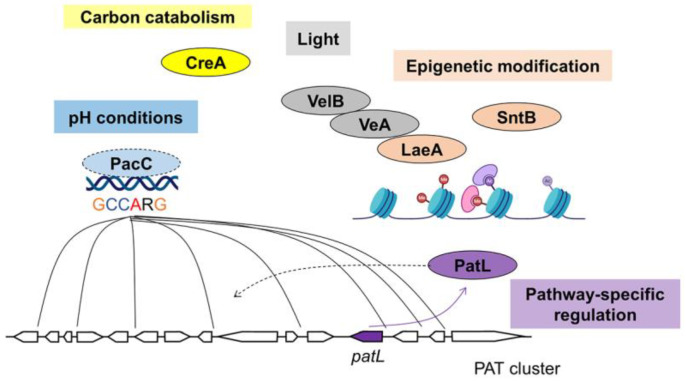
Regulatory mechanism of PAT biosynthesis.

PAT is produced by several fungal genera, including *Penicillium*, *Aspergillus* and *Byssochlamys* [82]. The *pat* BGC contains 15 genes, including a Zn(II)_2_Cys_6_ TF gene *patL*. PatL is found to be localized in the nucleus and acts as a pathway-specific regulator in *P. expansum* (Figure 3) [44,83]. No PAT was detected in a ∆*patL* mutant, and the *pat* genes were only marginally expressed in the ∆*patL* mutant [44]. The regulatory mechanism of how PatL regulates each *pat* gene is yet to be investigated but presumably will be operated similarly as AflR by binding to a specific motif in the promoters of other *pat* genes.

**Figure 4:**
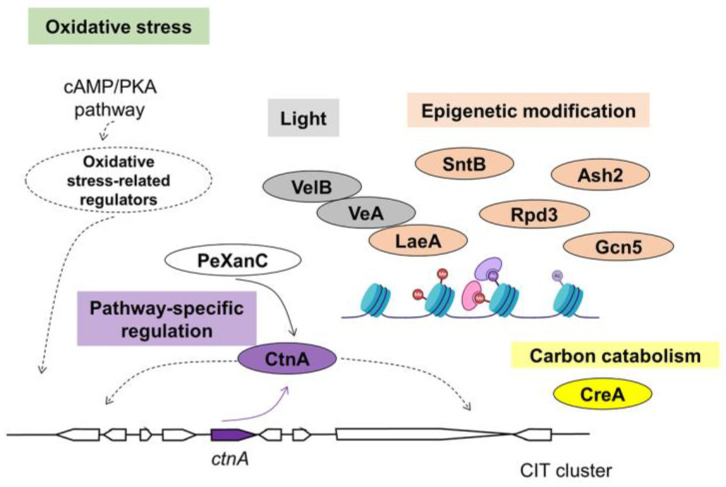
Regulatory mechanism of CIT biosynthesis.

CIT is mainly produced by *Penicillium*, *Aspergillus* and *Monascus* genera [26]. The reports of CIT biosynthesis are confusing and the known CIT clusters from *Penicillium* and *Monascus* species contain 6~9 genes [84,85,86]. He and Cox confirmed that CIT biosynthesis requires at least 6 genes by heterologous expression of the CIT biosynthetic genes in *A. oryzae* [19]. The Zn(II)_2_Cys_6_ TF CtnA (called Mrl3 in *M. ruber* M7) is conserved in some CIT clusters and functions as a pathway-specific regulator (Figure 4) [49,87]. Disruption of *ctnA* largely decreased the expression of polyketide synthase gene *citS* (also known as *pksCT*) and another gene *orf5*, and totally inhibited CIT production in *M. purpureus* [49]. Another report showed that the CIT product was reduced to 42% when replacing *ctnA* with *pks1* (a pigment-related gene) in *M. purpureus* [88]. In *P. expansum*, deletion of *ctnA* silenced expression of all of the other *cit* genes and resulted in loss of citrinin production [89]. Interestingly, *ctnA* is under regulation of another pathway-specific regulator in *P. expansum*, PeXanC. PeXanC acts in a trans fashion to induce expression of *ctnA* [89]. However, the transcription of *ctnA* is not totally dependent on PeXanC (Figure 4), demonstrating the complex regulatory network involved in CIT production.

**Figure 5:**
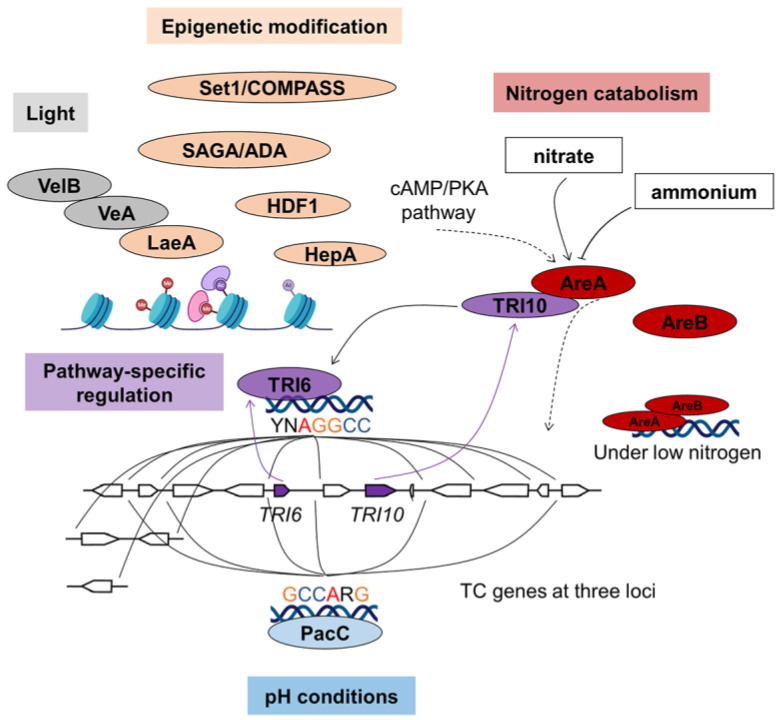
Regulatory mechanism of TC biosynthesis.

TCs are produced by *Fusarium* species fungi. The 15 trichothecene biosynthetic genes are found at three loci (Figure 5): the 12-gene core *TRI* cluster, the 2-gene *TRI1*–*TRI16* locus, and the single-gene *TRI101* locus [20]. In the *TRI* BGC, both *TRI6* and *TRI10* are positive regulator genes for TC biosynthesis. *TRI6* is a C2H2 type TF while *TRI10* is a protein without any known functional domains [55,90]. *TRI6* appears to have a larger effect than *TRI10*. Disruption of *TRI6* totally abolished the DON and T2-toxin biosynthesis [55]. The expression of nearly all the *TRI* genes (except *TRI10*) was reduced in ∆*TRI6* mutant, and the *TRI6* binding site 5′-YNAGGCC-3′ was found in the promoter regions of nearly all *TRI* genes (except *TRI10*) (Figure 5). Conversely, the expression of *TRI10* was significantly increased in ∆*TRI6* mutant, suggesting the transcription of *TRI10* is independent with *TRI6*. Disruption of *TRI10* abolished T2-toxin production and dramatically decreased the expression of four *TRI* genes (*TRI4*, *TRI5*, *TRI6* and *TRI101*). It is postulated that *TRI10* might act upstream of *TRI6* and is necessary for full expression of other *TRI* genes [90].

**Figure 6:**
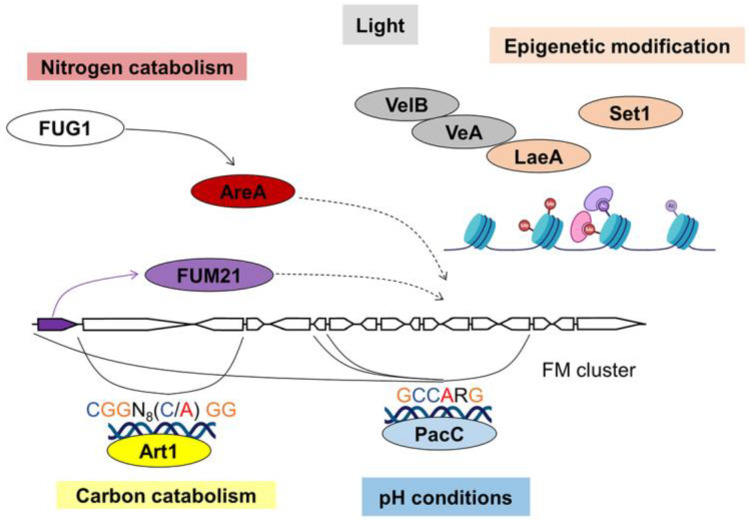
Regulatory mechanism of FM biosynthesis.

FMs are produced by species in *Fusarium*, *Aspergillus* and *Tolypocladium* genera [16]. The FM cluster consists of 17 genes (Figure 6), including a Zn(II)_2_Cys_6_ TF gene *FUM21* which functions as pathway-specific regulator. Deletion of *FUM21* reduced the expression of *FUM1* and *FUM8*, resulting in little to no FM production in *F. verticillioides* [66]. In *A. niger*, 10 out of 12 *FUM* genes were down-regulated in ∆*FUM21* mutant leading to loss of production of FM [91]. There is no report of a FUM21 DNA-binding site yet.

### 3.2. Global Regulators Response to Environmental Factors

Growing conditions usually have the most influence on the production of mycotoxins, and provide promising methods to control mycotoxin biosynthesis. Global regulators are often responsive to carbon and nitrogen source, pH, ambient light and oxidative stress [92]. This section reviews the connection between environmental factors and global regulators on mycotoxin synthesis.

#### 3.2.1. Carbon Source

The carbon source of growth media effects production of all characterized mycotoxins but the mechanism(s) of this regulation still remain cloudy. The C2H2 type TF CreA/Cre1 is the major transcriptional repressor of carbon catabolite metabolism in fungi but its role in mycotoxin synthesis is not consistent across fungal genera. Deletion of *creA* inhibited the production of AF in *A. flavus* (0.006 μg/g AF), while wild type (WT) strain and *creA* overexpression (OE::*creA*) strain produced about 0.096 μg/g and 0.105 μg/g respectively [33]. Although several *afl* genes have CreA-binding sites near their promoter regions, it appears that these sites are not active [93]. Recently the carbon responsive regulator RimO has been found to be required for *aflR* expression and ST production in *A. nidulans* but this gene is yet to analyzed in other mycotoxin producing fungi [34].

Studies of *P. expansum* have shown that glucose, maltose, fructose, mannose, sucrose and starch are favorable carbon sources for fungal growth, up-regulation *pat* gene expression and PAT production, while apple and citrus pectin, lactose, malic acid and cellulose were less favorable for growth with concomitant reduction in *pat* gene expression and PAT synthesis [94]. For CIT production, starch and saccharides reduced CIT level compared to rice flours, whereas brown rice flour enhanced CIT production significantly [95].

CreA loss in *P. expansum* reduces both PAT and CIT production but unexpectedly, *pat* genes were not down-regulated, but rather up-regulated in this mutant [45]. Indeed, a negative correlation was found between PAT accumulation and *creA* expression under sucrose-increasing content. Similarly, although CIT was not produced in ∆*creA*, *cit* genes were expressed [45]. The authors hypothesized that deletion of *creA* possibly impacted the availability of precursor pools required for PAT production and CIT production.

Studies of DON synthesis in *F. grainearum* showed that sucrose induces DON synthesis over glucose [96]. A role for CreA regulation of DON is not clear. Ten *TRI* genes, including *TRI6* and *TRI10*, contain a CreA binding site in their promoter regions but studies to determine if they are active have not been conducted. Furthermore, deletion of *creA* almost totally inhibits the growth of *F. graminearum*, thus obviating a clear route to focus on CreA impact on DON [97].

Currently there have not been any studies of any effects of CreA on FM biosynthesis although carbon source is important in laboratory studies. Sucrose is the preferred source to induce *FUM* gene expression and FM production over mannose and fructose, while glucose has no significant influence on the growth and FM production of *F. proliferatum* [98]. In addition, starch content in maize affects FM production and disruption of the α-amylase gene *AMY1* results in low levels of FM production [99]. A putative hexose kinase Hxk1 and a putative hexose transporter Fst1 have been demonstrated to be required for FM biosynthesis [100,101]. Further, a Zn(II)_2_Cys_6_ TF Art1, responsible for starch hydrolysis, might play a regulatory role in FM biosynthesis (Figure 6) [67]. The deletion strain produces no detectable FB_1_, and the putative Art1 DNA-binding sites (5′-CGGN_8_(C/A)GG-3′) have been found in the promoter regions of *FUM1* and *FUM7* [67].

#### 3.2.2. Nitrogen Source

Similar to carbon source, nitrogen source also affects production of all mycotoxins but not in a consistent manner. In some fungi, AreA, a GATA factor transcriptional regulator of nitrogen metabolism, has been deleted to explore impact on mycotoxin synthesis.

Different nitrogen sources impact AF production [102]. AreA was bound to the *aflR*/*aflS* intergenic region by recognizing a GATA element which seems to prevent AflR binding (Figure 2) [35,103]. The influence of AreA in AF biosynthesis was dependent on the nitrogen source media. In *A. flavus*, AFB_1_ production was reduced in ∆*areA* compared with WT strain in most conditions tested, but in Potato Dextrose Broth (PDB) medium the ∆*areA* strain promoted AF biosynthesis when compared with the WT and OE::*areA* strains [104]. As AreA itself is regulated by many other TFs (NmrA, MeaB, PnmB) dependent on media and environment, it is difficult to clearly outline a consistent role of AreA on AF synthesis (Figure 2).

In *P. expansum*, cultures grown with organic nitrogen sources give better PAT yields than inorganic nitrogen sources. Peptone, glutamic acid and yeast extract are the best nitrogen sources for up-regulation of all *pat* gene expression and increase PAT production in *P. expansum*, while ammonium sulfate is the most unfavorable nitrogen source [94]. But the regulatory mechanism between nitrogen and PAT biosynthesis is still unclear.

Organic nitrogen is also a better source for *Monascus* M9 growth and CIT production than inorganic nitrogen [95]. Minimal CIT production was observed in *M. purpureus* M3103 when grown with NH_4_Cl or NH_4_NO_3_ as the sole nitrogen source [105].

In *F. fujikuroi*, AreA and a second nitrogen metabolism regulator, AreB, have been found to regulate TC biosynthesis and TC production (Figure 5) [106,107]. AreA regulates the expression of some *TRI* genes by recognizing AreA binding sites in the promoter regions of *TRI6*, *TRI10* and other *TRI* genes [106]. In nitrogen-starving condition, AreB interacts with AreA to regulate TC production (Figure 5) [56].

In *F. verticilliodes*, the ∆*areA* mutant grows similarly to WT with the addition of ammonium phosphate, but FB_1_ was not produced under either low or high nitrogen levels in the ∆*areA* mutant [68]. Furthermore, *areA* was demonstrated to be down-regulated in the ∆*FUG1* mutant, an uncharacterized gene, and the production of FMs were reduced as well (Figure 6) [108]. It suggests that *FUG1* may affect FM biosynthesis through the nitrogen regulator AreA [108].

#### 3.2.3. pH

PacC (loss or reduction in phosphatase activity at acid but not at alkaline pH [Pac]) is the key TF in pH signal transduction in filamentous fungi, and recognizes 5′-GCCARG-3′ (R = G or A) in the target promoters [109]. The PacC cascade is activated under alkaline conditions and induces alkaline regulated genes while repressing acid regulated genes. Nitrogen source is important in pH regulation [110]. When ammonium sulfate is used as nitrogen source, assimilation of ammonia is associated with release of H^+^ cations, and will result in acidification of the medium [111].

Acidic conditions are more favorable for AF/ST biosynthesis, while AF/ST production is in low level in neutral and alkali media [36]. Lowering the pH to 4.0 in *A. flavus* resulted in increased AF production by 10-fold [112]. In *A. parasiticus*, a putative PacC binding site (5′-GCCAAG-3′) was identified in the *aflR* promoter (Figure 2), leading to the hypothesis that PacC could bind and repress the transcription of *aflR* under alkaline conditions [113].

Acidic conditions are also more favorable for PAT production than alkaline conditions, and pH 5.0 is the optimal condition [18,94]. *pat* gene expression and PAT production were reduced when pH was higher than 7.0 [94]. The growth of *P. expansum* presented a similar trend. Deletion of *pacC* had strikingly negative effects on *pat* gene expression and PAT production under both acidic and alkaline conditions, and also severely impaired growth and conidiation under both conditions. Besides, the PacC binding site (5′-GCCARG-3′) (R = G or A) was found in the promoter regions of 9 *pat* genes, including its putative pathway-specific regulator *patL* (Figure 3) [46]. It suggests that PacC is probably directly involved in regulating PAT biosynthesis although biochemical confirmation is currently not available.

In contrast to AF and PAT, CIT production was significantly increased when the pH value shift from acid to alkaline in *M. anka*, *P. citrinum*, *A. oryzae* and *A. niger* [114]. In *P. expansum*, more CIT was produced under higher pH conditions (pH 6~8) [85]. No report about the pH regulator PacC and CIT biosynthesis is available yet.

Acidic pH is a determinant of *TRI* gene transcription and TC production in *F. graminearum*. Neither *TRI* gene expression nor TC accumulation is detected when the pH is maintained at neutral or alkaline pH [115]. PacC represses *TRI* gene transcription and negatively regulates TC production. Overexpression of *pacC* in *F. graminearum* strongly repressed *TRI* gene expression and reduced TC accumulation at acidic pH [57]. Fourteen PacC binding sites are positioned in the promoter regions of 9 *TRI* genes, including the pathway-specific regulator *TRI6* (Figure 5) [116]. It indicates that PacC may regulate *TRI* cluster by directly binding to the promoters of some *TRI* genes.

FM biosynthesis is repressed by alkaline pH, but enhanced at acidic pH (3.0 to 4.0) [69]. Six *FUM* genes contain the PacC binding site in their promoter regions [69]. However, it is still not clear if *pacC* regulates FM biosynthetic genes by directly binding to their promoters.

#### 3.2.4. Light

**Figure 7:**
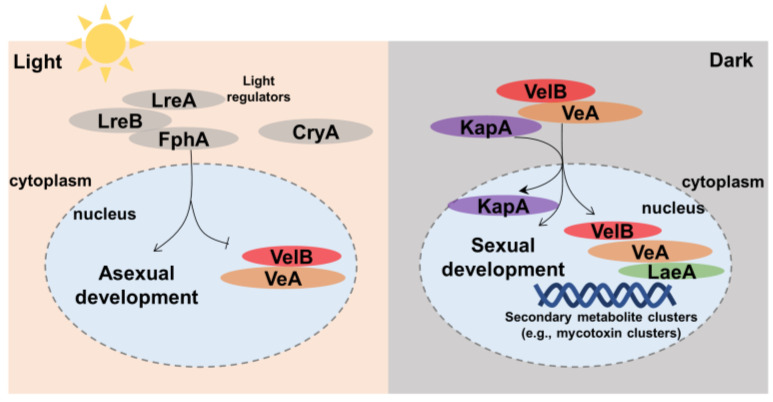
Velvet complex responses to light/dark condition and regulates mycotoxin biosynthesis.

Light response is strongly related to the “velvet complex” in filamentous fungi, and extensively investigated in *A. nidulans* [117]. The velvet family of regulators is known as a pivotal part in coordinating secondary metabolism (including mycotoxins) and differentiation processes in filamentous fungi [118]. The heterotrimeric velvet complex is a trimeric complex formed by three proteins: VelB-VeA-LaeA (Figure 7) [119]. It has been identified that *A. nidulans* develops asexually in light and sexually in the dark, and VeA is involved in the shift from sexual to asexual spore formation. LaeA is constitutively present in the nucleus, while VeA and VelB appear to interact already in the cytoplasm then travel together into the nucleus by KapA (Figure 7) [119]. The nuclear LaeA protein is a master regulator for multiple secondary metabolites including mycotoxins. The S-adenosyl methionine binding site of LaeA is critical for SM production [120]. No AF production is detected in ∆*veA* or ∆*laeA* strains in *A. flavus*, which is correlated with loss of AF BGC expression [37,38], and loss of both proteins also inhibits ST synthesis in *A. nidulans* [119].

The mechanism of Velvet complex response to light is highly conserved among filamentous fungi. In *P. expansum*, deletion of *veA*, *velB* and *laeA* inhibit PAT production, and consistently show down-regulated all 15 *pat* genes (Figure 3) [47,121]. On Potato Dextrose Agar (PDA) and Malt Extract Agar (MEA) medium, no CIT was detected in a ∆*veA* culture with decreased expression of all *cit* genes [50].

In *F. graminearum*, deletion of the velvet protein genes *veA* and *velB* reduced DON production [58,59]. The expression levels of the synthase gene *TRI5* and the pathway-specific regulator gene *TRI6* were decreased by 93% and 89%, respectively in ∆*velB* mutant [59]. Disruption of *laeA* resulted in a marked reduction in expression of 7 *TRI* genes, including *TRI6*, and abolished 15-ADON biosynthesis [60]. In *F. verticillioides*, loss of *lae1* (the *laeA* orthologue) reduced expression of all *FUM* genes. Surprisingly, despite decreased expression of *FUM* genes, FM production in the ∆*lae1* mutant was not significantly reduced compared with WT. However, the *lae1* complemented strain produced 50% more FMs than WT [122]. When the *veA* homologue was deleted in *F. verticillioides*, the production of FM was completely suppressed. VeA forms a complex with the velvet proteins VelB and VelC, and is necessary for the expression of the pathway-specific regulator gene *FUM21* (Figure 6) [70,71].

#### 3.2.5. Oxidative Stress

It is proposed that AF is part of the fungal oxidative stress response in *A. flavus* and *A. parasiticus* [123,124]. AtfB is a member of the bZIP/CREB family TF involved in oxidative stress. It has been demonstrated that AtfB binds on seven *afl* gene promoters by recognizing the cylic AMP-response element (CRE)-like site (Figure 2) [39]. The putative binding sites of another oxidative stress-related TF AP-1 have been found in the promoter region of *aflR* [40]. This information supports that AtfB and AP-1 may activate AF biosynthesis under high levels of oxidative stress-inducing conditions.

CIT is suggested as a protecting/antioxidative substance because an increase in the oxidative stress generated by H_2_O_2_ supplementation to the growth media leads to a concentration dependent increase in the production of CIT in *P. expansum* [125]. CIT could also protect against increased oxidative stress caused by increased Cu^2+^ concentrations and short wavelength light [126]. In addition, increasing amounts of external cAMP reduces CIT biosynthesis suggesting that a cAMP/PKA signaling pathway is involved in the regulation of CIT biosynthesis with respect to changes in the oxidative status of the fungal cell [51,126].

Functional or non-functional *TRI7* and *TRI13* genes lead to the production of different type of TCs, and *F. graminearum* is divided into two chemotypes: the DON chemotype and the NIV chemotype, for isolates producing DON/ADON or NIV/FX (Figure 1) [127,128]. The regulation of TCs by H_2_O_2_-induced oxidative stress is also chemotype dependent [129]. A 0.5 mM H_2_O_2_ stress increases DON/ADON production, while the same treatment inhibits NIV/FX production. But an opposite result was observed when treated with diamide. Whatever the chemotype is, the expression of TC biosynthesis was always strongly up-regulated during oxidative stress [130]. Fgap1 (Yap1 orthologue in *F. graminearum*) was shown to be involved in this regulation for both chemotypes. The NIV/FX chemotype has higher antioxidant capacities than DON/ADON chemotype in response to oxidative stress [130].

The effect of oxidative stress induced by H_2_O_2_ on FM production is dependent on *F. verticilliodies* isolate. Following the addition of H_2_O_2_, two *F. verticillioides* isolates increased FM production (>300%), while other three isolates produced significantly less (<20%) FM [131]. This is a key finding as most of the work described in this review focuses on single isolate of each species. It would be useful to determine if the regulatory characteristics is present across isolates of the same species.

### 3.3. Epigenetic Regulators

LaeA investigations first suggested that epigenetic regulatory mechanisms were important for secondary metabolism synthesis [119]. Since this initial work, dozens of studies have demonstrated that mycotoxin BGCs are subject to epigenetic regulation through the remodeling of chromatin. Histone modifying enzymes, such as histone acetyltransferases and methyltransferases, can place or remove post-translational modifications on histone tails which influence how tight or relaxed the chromatin is, impacting the transcription of mycotoxin BGCs [41]. This literature is vast and we cannot cover all of the studies but highlighted a few below and recommend the reader to refer to other reviews on this topic [132,133].

Deletion of the epigenetic reader gene *sntB* in *A. nidulans* and *A. flavus* changed the global levels of histone H3K9K14 acetylation, leading to the inhibition of ST and AF (Figure 2) [41,134], but induction of a silent secondary metabolite aspergillicin [135]. Most recently, it has been shown that SntB is part of a newly discovered chromatin binding complex known as the KERS complex, which like the Velvet complex, also links development to secondary metabolism [136]. Deletion of the histone acetyltransferase gene *rtt109* significantly decreased the production of AFs in *A. flavus* [42]. Deletion of the arginine methyltransferase gene *rmtA* in *A. flavus* decreased AFB_1_ production compared to the WT strain. RmtA also positively regulates the expression of *veA*. It is possible that RmtA regulates *afl* genes through the velvet protein VeA [43].

In *P. expansum*, deletion of *sntB* reduced expression level of the pathway-specific regulator gene *patL* and the polyketide synthase gene *patK*, and decreased PAT production in vitro and on apples. The expression of the CIT pathway-specific regulator gene *ctnA* and an oxidoreductase gene *citC* were also reduced, accompanied by decreased CIT production [48]. Moreover, the expression of *laeA*, *creA* and *pacC* was markedly down-regulated in the ∆*sntB* mutant. Although SntB has a wide effect on transcriptional complexes and TFs, deletion of *sntB* in *P. expansum* is not lethal [48].

In addition to SntB, CIT biosynthesis is also under the regulation of other epigenetic regulators (Figure 4). One is a histone H3K4 methyltransferase complex member Ash2. Lack of *ash2* gene resulted in loss of CIT production during 15 days of fermentation of *M. purpureus* [52]. Overexpression of the histone deacetylase encoding gene *rpd3* enhanced CIT production by more than 50%, with 6 key *cit* genes up-regulated in *M. ruber* [53]. Deletion of the histone acetyltransferase gene *gcn5* reduced CIT content to 21% of the WT strain in *M. ruber* [54].

Heterochromatin, histone methylation and acetylation also contribute to TC production in *F. graminearum* (Figure 5). Deletion of the heterochromatin protein gene *hepA*, reduced the H3K9me3 heterochromatic mark, and strongly decreased transcription of the synthase gene *TRI5* and the pathway-specific regulator gene *TRI6*, causing DON reduction, but did not affect the growth of *F. graminearum* [61]. Methyltransferase complex Set1/COMPASS has been found to catalyze H3K4 methylation in *Saccharomyces cerevisiae* [137]. Elimination of the histone modification by disrupting *Set1* abolished DON production in *F. graminearum*, with drastically decreasing the transcription levels of 8 *TRI* genes, including *TRI6* and *TRI10* [62]. Other two subunits involved in Set1/COMPASS, Bre2 and Sdc1, have been shown to physically interact with Set1 in regulating *TRI* genes [62]. The SAGA/ADA complex is responsible for the acetylation of H3K9, H3K18 and H3K27, and is also implicated in a regulatory role in DON induction [63]. Gcn5, SPT7 and ADA3 are all the components of the SAGA/ADA complex, and the deletion mutants all eliminate DON production. In addition, other two histone acetyltransferases, Sas3 and Elp3, responsible for H3K4 and H3K14 acetylation, also regulate the expression of *TRI* genes [63,64]. The histone deacetylase HDF1 also influence the production of DON [65].

There are fewer studies on the impact of epigenetic remodeling on FM. A methyltransferase of H3K4, Set1, showed a significant influence on FM biosynthesis and the expression of *FUM* genes [72]. Deletion of FgKMT5, a H4K20 methyltransferase, resulted in reduction of zearalenone production, another mycotoxin produced by *Fusarium* spp. [138].

## 4. Conclusions and Perspective

Mycotoxin contamination is a widespread hazard occurrence in foods and feeds. The internal regulation of mycotoxin biosynthesis is complex with variable environmental signals and regulators. Among the genetic regulators, pathway-specific regulators usually directly activate the target mycotoxin gene cluster. These pathway-specific regulators are impacted by both global and epigenetic regulators that respond to environmental cues.

Understanding the regulation of gene expression in mycotoxin biosynthesis helps to explain and develop control approaches by linking the environmental factors inducing toxin synthesis. For example, a treatment by a low-frequency (<300 Hz) magnetic field inhibits CIT contamination by reducing the expression of the pathway-specific regulator gene *ctnA* in *M. purpureus* [139]. Another potential strategy is to identify molecules that could inhibit pathway-specific regulators. Molecular docking methods, which are widely used in drug discovery, may enable the identification of novel antimycotoxinogenic molecules by predicting ligand-target interactions [140]. As most mycotoxin BGCs are induced or inhibited by other microbes, there remains potential to scale up screens with microbiome communities to look for inhibitory microbes that could be applied in biocontrol efforts. Regardless of any approach, there remains a need for intense efforts to develop future strategies for more effective methods to inhibit mycotoxin contamination.

## Figures and Tables

**Table 1:** Major mycotoxins and their fungal origin.

Mycotoxin	Species Producing	Reference
AFs and ST	*Aspergillus flavus*, *A. parasiticus*, *A. nomius*, *A. bombycis*, *A. pseudotamarii*, *A. toxicarius*, *A. parvisclerotigenus*, *A. ochraceoroseus*, *A. rambellii*, *Emericella astellata*, *E. venezuelensis*, *A. nidulans*, *A. versicolor*	[17,18,19]
PAT	*Penicillium expansum*, *P. griseofulvum*, *P. roqueforti*, *P. carneum*, *P. sclerotigenum*, *Alternaria alternata*, *Bysochlamis nivea*	[19,20]
CIT	*P. expansum*, *P. citrinum*, *P. verrucosum*, *P. radicicola*, *P. viridicatum*, *P. camemberti*, *Monascus purpureus*, *M. ruber*, *A. niger*, *A. terreus*, *A. oryzae*, *A. niveus*, *A. carneus*	[21,22]
TCs	*Fusarium graminearum*, *F. culmorum*, *F. cerealis*, *F. sporotrichioides*, *F. langsethiae*, *F. oxysporum*, *F. proliferatum*, *F. verticillioides*, *F. roseum*, *F. tricinctum*, *F.acuminatum*	[23,24]
FMs	*F. verticillioides*, *F. proliferatum*, *F. nygamai*, *F. napiforme*, *F. thapsinum*, *F. anthophilum*, *F. dlamini*, *F. moniliforme*, *Alternaria alternata*	[19,25]

**Table 2:** Summary of current known regulators involved in the regulation of mycotoxin biosynthesis.

	Regulators	Pathway-Specific Regulators	Global Regulators					Epigenetic Regulators
Mycotoxins			Carbon Source	Nitrogen Source	pH	Light	Oxidative Stress	
AFs		AflR [31], AflS [32]	CreA [33], RimO [34]	AreA [35]	PacC [36]	VelB-VeA-LaeA [37,38]	AtfB [39], AP-1 [40]	SntB [41], Rtt109 [42], RmtA [43]
PAT		PatL [44]	CreA [45]	N/A	PacC [46]	VelB-VeA-LaeA [47]	N/A	SntB [48]
CIT		CtnA [49]	CreA [45]	N/A	N/A	VelB-VeA-LaeA [50]	cAMP/PKA signaling pathway [51]	SntB [48], Ash2 [52], Rpd3 [53], Gcn5 [54]
TCs		TRI6, TRI10 [55]	N/A	AreA, AreB [56]	PacC [57]	VelB-VeA-LaeA [58,59,60]	N/A	HepA [61], Set1/COMPASS [62], SAGA/ADA complex (Gcn5, SPT7, ADA3) [63], Sas3, Elp3 [64], HDF1 [65]
FMs		FUM21 [66]	Art1 [67]	AreA [68]	PacC [69]	VelB-VeA-LaeA [70,71]	N/A	Set1 [72]

N/A: not available.

## Data Availability

Not applicable.
